# Positive Predictive Value of High-Grade Prostate Imaging and Reporting Data System V2.1 Magnetic Resonance Imaging Findings for Prostate Cancer

**DOI:** 10.4314/ejhs.v34i1.5S

**Published:** 2024-10

**Authors:** Abdudin Heru Mehammed, Alemayehu Bedane Worke, Ashenafi Aberra Buser, Semira Abrar Issa, Takele Menna, Tesfaye Kebede Legesse, Assefa Getachew Kebede

**Affiliations:** 1 Department of Radiology and Medical Radiologic Technology, School of Medicine, St. Paul's Hospital Millennium Medical College, Addis Ababa, Ethiopia; 2 Department of Radiology, School of Medicine, Addis Ababa University, Addis Ababa, Ethiopia; 3 Department of Public Health, School of Public Health, St. Paul's Hospital Millennium Medical College, Addis Ababa, Ethiopia

**Keywords:** Prostate Imaging and Reporting Data System (PI-RADS), Prostate Cancer, Prostate MRI Findings

## Abstract

**Background:**

Prostate cancer is a leading cause of cancer-related mortality among men, second only to lung cancer. Prostate magnetic resonance imaging (MRI) utilizing the Prostate Imaging and Reporting Data System (PI-RADS) v2.1 scoring system effectively stratifies patients by risk and correlates significantly with histopathological outcomes. This study aimed to assess the positive predictive value (PPV) of high-grade PI-RADS v2.1 MRI findings and their correlation with histopathological results from biopsies in patients visiting the interventional radiology unit at St. Paul's Hospital Millennium Medical College (SPHMMC).

**Methods:**

A facility-based cross-sectional study was conducted involving patients referred to the SPHMMC interventional radiology unit with high-grade PI-RADS v2.1 MRI findings who underwent TRUS-guided prostate biopsy between January 2023 and April 2024.

**Results:**

Among 105 patients, the PPV was 94.5% for a PI-RADS v2.1 score of 5 and 51.5% for a score of 4. These findings underscore the predictive power of high-grade PI-RADS scores, particularly for score 5 lesions, aiding clinicians in decision-making for further investigations and treatment. Significant correlations were observed between MRI characteristics—such as ill-defined margins, larger size, and extraprostatic extension— and high-grade PI-RADS scores in the peripheral zone (p<0.01).

**Conclusion:**

High-grade PI-RADS v2.1 scores exhibit strong positive predictive value for detecting prostate cancer, emphasizing the essential role of multiparametric MRI in diagnosis. Integrating multiparametric MRI findings with clinical and laboratory data can further enhance patient care and outcomes.

## Introduction

Prostate cancer ranks among the most prevalent malignancies affecting men globally. Given its high incidence, there is a pressing need for improved diagnostic and treatment strategies(1). Malignant lesions are primarily located in the peripheral zone (PZ), with a smaller proportion in the transitional zone (TZ)(2). Lesions in the TZ often appear lenticular-shaped, while benign prostatic hyperplasia (BPH) typically presents as round or oval. Conversely, cancers in the PZ generally exhibit round or oval shapes, contrasting with the wedge or linear appearances associated with benign cases (3).

Malignant tumors are often larger, hypointense on T2-weighted imaging, and present lower mean ADC(Apparent diffusion coefficient) values(4). They may also demonstrate early enhancement in dynamic contrast-enhanced MRI(**Magnetic Resonance Imaging**), although there can be considerable overlap in imaging features, especially in the TZ(5,6).

Historically, prostate cancer diagnosis relied on ultrasound, laboratory PSA(Prostate-specific antigen) levels, and finger-guided biopsies. However, advanced imaging techniques, particularly MRI using the PI-RADS(***Prostate Imaging and Reporting Data System***) v2.1 protocol, have transformed diagnostic and management approaches(7). Higher PI-RADS scores correlate with greater Gleason scores and clinically significant prostate cancer, alongside poor prognostic indicators like extracapsular extension and lymphovascular invasion(8).

A meta-analysis of 17 studies revealed a cancer detection rate of 59% for a PI-RADS score of four and 85% for a score of five, indicating a strong relationship between higher scores and detection rates(9). The diagnosis of prostate cancer is challenging due to its heterogeneous features and variable clinical behavior, necessitating reliable tools(1).

PI-RADS v2.1 has enhanced the standardization and reproducibility of prostate imaging, incorporating multiparametric MRI techniques—including T2-weighted imaging, diffusion-weighted imaging (DWI), and dynamic contrast-enhanced sequences—to significantly boost diagnostic accuracy(8,10,11).

In Ethiopia, prostate cancer represents a significant health burden, accounting for a notable percentage of cancer cases and deaths(12). This study aims to evaluate the positive predictive value of highly suspicious PI-RADS v2.1 MRI findings by correlating them with histopathological results.

## Materials and Methods

**Study area and design**: This study was conducted at St. Paul's Hospital Millennium Medical College (SPHMMC) in Addis Ababa, a tertiary referral and teaching hospital. The interventional radiology unit frequently performs TRUS(Transrectal Ultrasound)-guided prostate biopsies, primarily for PI-RADS 4 and 5 lesions.

A cross-sectional study design was employed, reviewing all patients with high-grade PI-RADS v2.1 lesions on mpMRI(***multiparametricMRI***) during the study period. Multiparametric prostate MRI was performed using a 1.5 Tesla MRI machine.

**Source and study population**: An exhaustive sampling method included all patients who underwent TRUS-guided prostate biopsy between January 2023 and April 2024, (160 patients in total). Inclusion criteria encompassed adult males with prostate MRI and TRUS-guided biopsy results at SPHMMC during the study period. Patients with suboptimal MRI quality or a history of prostatectomy were excluded, resulting in a final sample of 105 patients.

**Variables**: The independent variables included age, lesion location, T2-weighted signal intensity, shape, margin, size, DWI/ADC characteristics, DCE enhancement pattern, and extraprostatic extension. The dependent variable was the histopathological result (benign or prostate cancer).

### Operational definitions

High-Grade and Very High-Grade Prostate Lesions: Defined as PI-RADS v2.1 scores of four and five, respectively(13).

Diffusion restriction: Indicated by high DWI signal corresponding to low ADC signal(5).

Dynamic Contrast-Enhanced (DCE) MRI: Positive DCE is noted with focal and timely enhancement of suspicious lesions relative to normal tissue(13,14).

**Data collection procedure**: Multiparametric prostate MRI reports were accessed via the radiology information system, and histopathology results were retrieved from the pathology department. The mpMRI was conducted using Siemens 1.5T MRI, adhering to standard protocols, with data collected by the principal investigator and reviewed by an experienced radiologist.

**Data analysis**: Data were coded and analyzed using Epi Info and SPSS. Descriptive statistics summarized categorical variables, and chi-square and Fisher exact tests assessed significant differences between imaging descriptors and histopathological results (p≤0.05).

**Ethical clearance**: Ethical approval was obtained from SPHMMC's research review board. Anonymity, privacy, and confidentiality were strictly maintained throughout the study.

## Results

**Study population characteristics**: Out of the 160 patients who underwent TRUS-guided prostate biopsy between January 2023 and April 2024, 105 were eligible for inclusion. Participants' ages ranged from 46 to 92 years, with the majority (67.6%) being between 61 and 80 years old. Most patients had a prostate volume ranging from 51 to 100 cc, a PSA value between 20 and 50 ng/dl, and a PSA density greater than 0.21 (Table 2).

**Table 2:** Socio-demographic and general information of participants along with mean and median value after objective normality test with p-value was done **(n=105)**

Variables	Range	Frequency(proportion)	Mean (SD)
Age (yr.)	41-60	24(22.9)	67.85 (9.58)
	61-80	**71(67.6)**	
	>=81	10(9.5)	
			**Median (IQR)**
Prostate volume	<=50	26(24.8)	68 (50, 96)
(cm^3^)	51-100	**56(53.3)**	
	101-150	13(12.4)	
	>=151cm^3^	10(9.5)	
PSA value(ng/dl)	4-10-20	9(8.6)	34.7 (18, 62.6)
	10-20	20(19)	
	20-50	**44(41.9)**	
	>50	32(30.5)	
PSA density	<=0.1	5(4.8)	0.46 (0.26, 0.7)
	0.11-0.15	7(6.7)	
	0.151-0.2	10(9.5)	
	>=0.21	**83(79)**	

Among the 105 mpMRI findings with PI-RADS v2.1 scores of 4 and 5, 78 (74.3%) were found in the peripheral zone (PZ), while 27 (25.7%) were located in the transition zone (TZ). Of the 78 lesions in the PZ, 73 (93.5%) were prostate cancer, while the remaining were benign. In contrast, only 12 (44.4%) of the 27 lesions in the TZ were malignant.

**Outcome measurement**: Among the 105 patients who underwent TRUS-guided prostate biopsy, prostate cancer was detected in 81% (95% CI: 72.1-87.9) of cases. The remaining 19% (95% CI: 12.1-27.9) had benign conditions, such as benign prostatic nodules and prostatitis.

Of all patients, 72 (68.6%) had a PI-RADS v2.1 score of 5, while 33 (31.4%) had a score of 4. Among those with a PI-RADS v2.1 score of 5, 68 patients were diagnosed with prostate cancer, resulting in a positive predictive value (PPV) of 94.5%). Among the 33 patients with a PI-RADS v2.1 score of 4, 17 were diagnosed with prostate cancer, yielding a PPV of 51.5% (Table 3). The 20 benign prostate lesions identified included 12 cases (60%) of prostatitis and 8 cases (40%) of nodular hyperplasia. Nodular hyperplasia was exclusively found in the TZ, while prostatitis was present in both zones, with 58% of cases occurring in the PZ.

**Table 3:** Assessment of the positive predictive value of high-grade PI-RADS version2.1 scores and comparison with post-biopsy histopathology outcome (n=105)

Final PI-RADS version 2.1 score Vs Histopathology Results cross-tabulation	Histopathology results			

		Prostate cancer (Positive predictive value)	Benign	Total
Final PI-RADS v2.1 score	4	17(51.5%)	16(48.5%)	33
	5	68(94.5%)	4(5.5%)	72
Total		85	20	105

**Correlation between MRI Imaging findings of High-Grade prostate lesions and histopathological results**: The results of the Chisquare and Fisher exact tests revealed significant correlations between the presence of prostate cancer and various PI-RADS v2.1 imaging descriptors, including lesion location, shape, appearance on DWI/ADC maps, DCE characteristics, presence of extraprostatic extension, size, margin, and overall PI-RADS v2.1 score.

The majority of lesions located in the peripheral zone were prostate cancer compared to those in the transitional zone (93.5% vs. 44.4%, p < 0.01). This significant p-value indicates a strong statistical difference between the two zones, emphasizing lesion location as a critical factor in determining the likelihood of malignancy. Similarly, lesions with lenticular shapes had a higher chance of being prostate cancer compared to those with wedge-shaped morphology (p < 0.01).

Lesions with extraprostatic extension into adjacent tissue had a high likelihood of being prostate cancer, as all identified cases were confirmed as malignant (p = 0.02). This suggests that extraprostatic extension is a crucial indicator of disease severity and prognosis.

Lesion size also significantly correlated with the presence of prostate cancer; lesions measuring 1.5 cm or larger were more frequently identified as malignant compared to those smaller than 1.5 cm (94.6% vs. 46.6%, p < 0.01). The presence of larger prostatic lesions should alert clinicians to the possibility of prostate cancer and the need for further testing, monitoring, and treatment.

In the Chi-square test correlating lesion margins with prostate cancer, failure was detected, prompting the use of Fisher's exact test. This revealed that lesions with ill-defined margins were more likely to be prostate cancer compared to those with well-circumscribed borders (83.3% cancer vs. 14.9% benign, p = 0.006). The final PI-RADS v2.1 scores were also correlated with the presence of prostate cancer. Lesions with a score of 5 had a significantly higher likelihood of being malignant than those with a score of 4 (94.5% vs. 51.5%, p < 0.01). Similarly, lesions with diffusion restriction and positive DCE had a greater chance of being prostate cancer compared to those with facilitated diffusion and negative DCE (both p < 0.05) (Table 4). Lesions with hypointense signal intensity did not show a significant correlation with malignancy when compared to other signal intensities (p = 0.39).

**Table 4:** mpMRI PIRADS v2.1 findings (T2w, margin, Shape, DWI/ADC map, DCE, extraprostatic extension, size, and location) of the lesion, final P-IRADS score and comparison between prostate cancer Vs benign histopathology outcome (n = 105)

	Histopathological outcome			P-value
		
	Prostate cancer=n (%)	Benign=n (%)	Total=n (%)	
**Margin of the lesion**				
Well-circumscribed	5(45.5)	6(54.5)	11(10.5)	**0.006**
Ill-defined	80(83.3)	14(14.9)	94(89.5)	
**The shape of the lesion**				
Lenticular	55(98.2)	1(1.8)	56(53.3)	**<0.01**
Wedge	7(63.6)	4(36.4)	11(10.5)	
Round/oval	23(60.5)	15(39.5)	38(36.2)	
**DWI/ADC maps**				
Restricted	85(83.3)	17(16.7)	102(97.1)	**0.006**
Facilitated	0(0)	3(100)	3(2.9)	
**DCE findings**				
Positive	66(97)	2(3)	68(64.8)	**<0.01**
Negative	19(51.3)	18(48.7)	37(35.2)	
**Extra-prostatic extension**				
Yes	29 (100)	0(0)	29(27.6)	**0.02**
No	56(73.6)	20(26.4)	76(72.4)	
**Size of the lesion**				
1.49cm and less	14(46.6)	16(53.4)	30(28.6)	**<0.01**
1.5 and more	71(94.6)	4(5.4)	75(71.4)	
**Location of the lesion**				
Transitional zone	12(44.4)	15(55.6)	27(25.7)	**<0.01**
Peripheral zone	73(93.5)	5(6.5)	78(74.3)	
**T2w findings**				
Hypointense	67(82)	14(18)	81(77%)	0.39
Mixed/Heterogeneous	18(75)	6(25)	24(23%)	
**PI-RADS v2.1 final score**				
4	17(51.5)	16(48.5)	33(31.5)	**<0.01**
5	68(94.5)	4(5.5)	72(68.5)	

## Discussion

This study, the first of its kind in the country and likely reflects the tendency of patients in our setting to present with more advanced disease, underscoring the importance of early detection and region, demonstrated a positive predictive value of 94.5% for lesions with a PI-RADS v2.1 score of 5 and 51.5% for those with a score of 4. Comparisons with studies conducted in China and Germany revealed that our prostate cancer detection rate for PI-RADS v2.1 score 4 was comparable, while our detection rate for score 5 was slightly higher than in Germany(9,15). This increased detection rate screening for prostate cancer.

In assessing the distribution of prostate cancer lesions, 74.3% were located in the PZ, consistent with previous studies reporting similar distributions (PZ: 70%, TZ: 30%)(2). However, a meta-analysis from Stanford University reported a higher percentage of lesions in the PZ (82%)(16).

Our findings also indicated a significant correlation between lesion size (1.5 cm or larger) and the likelihood of a prostate cancer diagnosis, corroborating prior research linking larger lesions to increased malignancy risk(4,17).

Among the 85 prostate cancer cases identified, the majority (64.7%) exhibited a lenticular or crescentic shape, while 27% were round or oval. This contrasts with a Canadian study focusing on lesion shapes in the PZ, which reported a higher proportion of round or oval lesions (63.9%)(18). This discrepancy may stem from differences in study design, as our study included lesions from all zones.

The analysis of benign histopathological outcomes revealed that prostatitis was most common in the PZ, while nodular hyperplasia was exclusively found in the TZ. The occurrence of nodular hyperplasia in the TZ is attributed to unregulated hyperplastic growth of the epithelial and fibromuscular tissues. These benign conditions can mimic prostate cancer, emphasizing the need for accurate diagnosis through detailed mpMRI features and histopathological studies(19).

Our study confirmed significant differences in the presence of benign lesions versus prostate cancer among PI-RADS v2.1 scores of 4 and 5, aligning with previous research(8,20). The association between ill-defined margins, larger size, and extraprostatic extensions with prostate cancer presence was also supported by studies in diverse populations(2,4,16,21,22).

In conclusion, the study demonstrated that PI-RADS v2.1 scores of 4 and 5 are reliable indicators of malignancy, consistent with established literature. Various MRI characteristics—such as dynamic contrast enhancement, lesion size, PZ location, ill-defined margins, diffusion restriction, and extraprostatic extensions—were significantly associated with positive histopathology results, enhancing non-invasive evaluation and classification of prostate cancer risk. These findings can inform biopsy decisions and improve patient outcomes.

The limitations of the study include the focus solely on patients with PI-RADS v2.1 scores of 4 and 5, which restricted the analysis to positive predictive value. Other metrics, such as sensitivity, specificity, and negative predictive value (NPV), were not assessed. The retrospective design presents challenges, including potential gaps in recorded data and control for confounding variables. Additionally, MRI findings and histopathology results were reported by different physicians, potentially introducing inter-reader variability.

Despite these limitations, the findings support the integration of the PI-RADS v2.1 scoring system with clinical and laboratory data-such as PSA levels and DRE exams-to enhance patient management decisions. Given the high PPV of PI-RADS 5, this scoring system should be adopted in routine clinical practice for prostate cancer diagnosis in Ethiopia, particularly for patients presenting with advanced disease.

The clinical implications of this study are significant, especially regarding screening and diagnosis of prostate cancer in Ethiopia, where incidence rates are rising. Accurate lesion stratification using PI-RADS v2.1 will facilitate early identification and management, leading to better patient care, optimized resource utilization, and improved morbidity and mortality outcomes. While the PI-RADS scoring system relies on advanced imaging technology, the increasing availability of MRI machines across regional and zonal cities makes standardization advisable. This standard application will foster a universal language among radiologists and urologists.

Further research is encouragedneeded to explore factors influencing the predictive diagnostic capability of the PI-RADS v2.1 scoring system. Studies utilizing advanced MRI techniques and comprehensive evaluations of sensitivity, specificity, and negative predictive value should employ larger sample sizes and multicentric, prospective designs.

## Figures and Tables

**Table 1:** Reference for PI-RADS v2.1 category 4 and 5 (13)

mpMRI sequence	PI-RADS v2.1 category	Location In the peripheral zone	Location in the transitional zone
T2 weighted imaging	4	Circumscribed, homogenous moderate hypointense focus/mass confined to the prostate and <1.5 cm in greatest dimension.	Lenticular or non-circumscribed, homogeneous, moderately hypointense, and <1.5 cm in greatest dimension.
	5	Same as 4 but >1.5cm in greatest dimension or definite extraprostatic extension/invasive behavior	Same as 4, but ≥ 1.5cm in greatest dimension or definite extraprostatic extension/invasive behavior.
Diffusion-weighted imaging	4	Focal markedly hypointense on ADC and markedly hyperintense on high b-value DWI; <1.5cm in greatest dimension	Focal markedly hypointense on ADC and markedly hyperintense on high b-value DWI; <1.5cm in greatest dimension
	5	Same as 4 but ≥1.5cm in greatest dimension or definite extraprostatic extension/invasive behavior	Same as 4 but ≥1.5cm in greatest dimension or definite extraprostatic extension/invasive behavior
